# Is There Any Point in a Corticosteroid Treatment of Intermittent Asthma?

**DOI:** 10.1100/tsw.2007.134

**Published:** 2007-07-03

**Authors:** Ivana Stanković, Tatjana Pejčić, Branislava Milenković, Dragana Jovanović, Milan Rančić

**Affiliations:** ^1^ The Clinic for Lung Diseases and TBC, University Hospital, Niš, Serbia; ^2^ The Institute for Lung Diseases, University Hospital, Belgrade, Serbia

**Keywords:** intermittent asthma, bronchial hyperresponsiveness, inhaled corticosteroid

## Abstract

International guidelines advocate the early introduction of inhaled corticosteroids (ICS) in all types of persistent asthma. Our study was undertaken to assess the effects of ICS on bronchial hyperresponsiveness (BHR) as a hallmark of inflammation, and to assess the symptoms, the use of rescue medications, and the parameters of lung function in patients with mild intermittent asthma. The patients with intermittent asthma (n = 85) were randomly allocated to a treatment with ICS, beclomethasone dipropionate 250 μg/day and short-acting β2 agonists salbutamol as needed (Group A, n = 45) or to a treatment with only short-acting β2 agonists as needed (Group B, n = 40) during the 6-month treatment period. At the end of the study, in Group A, we found a statistically significant decrease of BHR (PD_20_ 0.98 vs. 2.07) (p < 0.001), diurnal peak expiratory flow (PEF) variability (17.9 vs. 13.8) (p < 0.001), symptom scores (0.63 vs. 0.30) (p < 0.001), and used rescue medication (p < 0.001), while the parameters of lung function remained unchanged except for forced expiratory volume in 1 sec (FEV1), which had a statistically significant increase (3.58 vs. 3.66) (p < 0.001). In Group B, there was a statistically significant decrease of lung function parameters FEV1 (3.80 vs. 3.71) (p < 0.001), forced vital capacity (FVC) (4.43 vs. 4.37) (p < 0.001), FEV1/FVC (88.2 vs. 85.3) (p < 0.05), PEF (8.05 vs. 7.51) (p < 0.01), PEF variability (17.85 vs. 18.33) (p < 0.001), increased BHR (PD_20_ 1.04 vs. 0.62) (p < 0.05), and symptom scores (0.46 vs. 0.62) (p < 0.01), as well as the use of rescue medication during the day (p < 0.001). Early introduction of low doses of ICS may be more beneficial than β2 agonists alone in patients with intermittent asthma.

### INTRODUCTION

Asthma is a disease that is characterized by airway inflammation and bronchial hyperresponsiveness (BHR). Mediators released by inflammatory cells, mainly eosinophils and mast cells, may injure airway tissue and lead to a remodeling of the airways, an airflow obstruction, and an increase in BHR with recurrent episodes of wheezing, coughing, and shortness of breath[1]. Remodeling represents a dynamic process that occurs in reaction to an inflammatory insult, and it results in changes in the composition, content, and organization of the cellular and molecular constituents of the airway wall in asthma. It is now believed that remodeling may occur earlier in the disease process than originally thought. In their study, Vignola et al. found that the basement membrane size was significantly increased in patients with mild intermittent asthma in comparison with the control subjects[2]. The data suggest that airway remodeling occurs even in mild intermittent asthma. Recent data indicate that the early use of anti-inflammatory therapy might limit airway remodeling and may modify the disease process[3]. The corticosteroids are considered the most potent and consistently effective long-term control medication in asthma.

Asthma treatment per the Global Initiative for Asthma (GINA) recommends that patients with persistent asthma should use an inhaled corticosteroid (ICS) as the first-line maintenance treatment. However, patients with mild intermittent asthma are advised to use a short-acting β2 agonist bronchodilator as needed, without the use of controller medication[1]. Taking into consideration the fact that inflammation, the process of remodeling, BHR, and the symptoms of disease are present in patients with intermittent asthma, our study aimed at analyzing the impact of the use of ICS on BHR in patients with mild intermittent asthma, because the degree of BHR is considered to be indirectly related to the degree of bronchial inflammation, the symptoms of the disease, and the parameters of lung function.

### METHODS

The study was performed as a prospective, randomized, parallel group trial in the outpatient departments of the Lung Disease Clinic. We studied 96 patients with mild intermittent asthma who were selected for the study according to GINA, on the functional criteria for mild intermittent asthma, and who had a documented need of short-acting β2 agonists for relief of asthma symptoms, maximally two doses per week for at least a year. The patients were randomly allocated to the treatment with ICS, beclomethasone dipropionate 250 μg/day and short-acting β2 agonists (Ventolin) as needed rescue medication (Group A) or to the treatment with only short-acting β2 agonists as needed (Group B) during the 6-month treatment period. Eleven subjects did not stay until the end of the study (eight did not regularly attend the clinic and three moved away). Therefore, 45 patients in Group A and 40 in Group B completed the study.

On enrollment, all the patients kept a diary card and recorded morning and evening peak expiratory flow (PEF) values (best of three measurements), asthma symptoms, and the use of short-acting β2 agonists during the run-in period (2 weeks) and during the last 2 weeks of examination. The number of as-needed inhalations of short-acting β2 agonists was recorded in the diary every evening covering the previous 24-h period.

At the beginning and the end of the study, the spirometry and bronchoprovocative test with histamine were measured in all the patients. The skin prick test was performed in order to examine the atopic status.

### Spirometry

The parameters of lung function: forced expiratory volume in 1 sec (FEV1), forced vital capacity (FVC), and FEV1/FVC×100 were recorded using flow-volume spirometer (Jaeger Masterlab) according to the recommendations of the European Respiratory Society[4].

PEF was measured twice a day by a Mini Wright peak flow meter; diurnal PEF variability was calculated according to the formula: (evening-morning PEF/the mean of the evening and morning PEF) × 100 and the results shown as the mean diurnal variability.

### Bronchoprovocative Test

Aerosol of histamine was delivered by a dosimeter device (APS). The challenge started with a histamine dose of 15 μg, which was then increased in doubling doses until FEV1, measured within 1 min of histamine inhalation, decreased 20% or more when compared with the baseline of FEV1. The dose of histamine causing a 20% decrease in FEV1 (PD20 histamine) was calculated by interpolation of the dose-response curves[5,6].

### Skin Tests

The atopic status was assessed by the skin prick test to a panel of five common aeroallergens and was defined as positive of ≥3 mm to one or more allergens.

### Daily Asthma Symptom Score

Four asthma symptoms (daytime breathlessness, daytime wheeze, daytime cough, and nighttime asthma) were scored by the patients every day using a subjective 0–3 score system where 0 = none, 1 = mild, 2 = moderate, 3 = severe. The symptoms were added each day and a mean weekly score calculated for the period of assessment.

### Statistical Analysis

Data were analyzed in the methodology of descriptive and analytic statistics. The adequate parametric and nonparametric statistical tests were used: Student's t test, Mann-Whitney U test, Pearson's X2 test, Fisher's test correct probability, Wilcoxon's test for matched pairs.

## RESULTS

Table 1 shows the baseline demographic characteristics of all the participants. The groups were comparable and there was no significant difference in terms of age, sex, atopic status, and smoking characteristics. Thus, 45 subjects in Group A (28 female, 17 male; mean age 35.04 ± 8.24 years) treated with ICS, beclomethasone dipropionate 250 µg/day and short-acting β2 agonists (Ventolin) as needed, and 40 subjects in Group B (25 female, 15 male; mean age 34.15 ± 8.31) treated according to GINA with only short-acting β2 agonists as needed, completed the 6-month trial.

**TABLE 1**
**Demographic Characteristics of the Patients**

| | Group A (n = 45) | Group B (n = 40) |
|---|---|---|
| Age | 35.4 ± 8.24 | 34.15 ± 8.31 |
| Sex (M/F) | 17/28 | 15/25 |
| Atopic | 73% | 79% |
| Smoking | | |
| Never | 77.7% | 80% |
| Ex-smoker | 22.3% | 20% |
| Current | 0% | 0% |

At the beginning of the study, FEV1, FVC, and FEV1/FVC were similar in both groups. After 6 months, at the end of the study, in Group A there was a statistically significant improvement of FEV1

3.58 vs. 3.66 ($p < 0.001$); although FVC, FEV1/FVC, and PEF were improved, the change was not statistically significant. In Group B, there was a statistically significant decrease of all the analyzed spirometric parameters: FEV1 3.80 vs. 3.71 ($p < 0.001$), FVC 4.43 vs. 4.37 ($p < 0.001$), FEV1/FVC 88.2 vs. 85.3 ($p < 0.05$), and PEF (8.05 vs. 7.51) ($p < 0.01$). After having compared the parameters of lung function between Groups A and B at the end of study, we did not find statistically significant differences.

At the end of the study, diurnal PEF variability was significantly decreased in Group A 17.9 vs. 13.8 ($p < 0.001$) and significantly increased in Group B 17.85 vs. 18.33 ($p < 0.001$). When Groups A and B were compared at the end of the study, there was a statistically significant difference PEF variability ($p < 0.001$). The changes in the parameters of lung function are shown in Table 2.

**TABLE 2**
**Lung Function, Initially and at the End of the Study**

| | Group A | | Group B | |
|---|---|---|---|---|
| | Baseline | End of Study | Baseline | End of Study |
| FEV1 (L) | 3.58 ± 0.89 | 3.66 ± 0.88* | 3.80 ± 0.86 | 3.71 ± 0.82* |
| FVC (L) | 4.21 ± 1.03 | 4.25 ± 1.04 | 4.43 ± 1.01 | 4.37 ± 0.98* |
| FEV1/FVC | 87.3 ± 6.26 | 87.1 ± 4.55 | 88.2 ± 6.97 | 85.3 ± 5.27*** |
| PEF (L/s) | 8.06 ± 2.42 | 7.84 ± 1.78 | 8.05 ± 1.88 | 7.51 ± 1.54** |
| PEF var(%) | 17.9 ± 0.92 | 13.81 ± 1.45* | 17.85 ± 0.82 | 18.33 ± 1.19*,[a] |

*$p < 0.001$, **$p < 0.01$, ***$p < 0.05$, [a]$p < 0.001$(Group A vs. B at the end of study).

At the end of the study there was a high, significant improvement in reduced BHR in Group A ($PD_{20}$ 0.98 vs. 2.07) ($p < 0.001$) and a significant increase in untreated group B ($PD_{20}$ 1.04 vs. 0.62) ($p < 0.05$) (Table 3). The analysis of BHR in relation to atopic status shows that at the beginning of the examination in both groups, there was not a significant change in relation to the atopic status. At the end of the examination, in Group A, both in the atopic ($PD_{20}$ 0.92 vs. 2.01) ($p < 0.001$) and the nonatopic patients ($PD_{20}$ 1.14 vs. 2.21) ($p < 0.05$), there was a significant decrease of BHR. In Group B, there was a significant increase of BHR ($PD_{20}$ 1.04 vs. 0.64) ($p < 0.001$) in the atopic patients, while in the nonatopic, there was an increase of BHR, but it was not significant (Table 4). In Group A, there was a significant reduction of the requirements for inhaled bronchodilator in the last 2 weeks in comparison with the baseline: day use 1.17 ± 0.24 puffs/week were reduced to 0.51 ± 0.18 ($p < 0.001$) and night use 2.51 ± 0.76 puffs/month were reduced to 0.58 ± 0.69 ($p < 0.001$). Short-acting β2 agonists were used much more frequently during the day, 0.86 vs. 1.15 puff ($p < 0.001$), in Group B at the end of examination (Table 5). Daily asthma symptom scores were significantly reduced in Group A (0.63 vs. 0.30) ($p < 0.001$) and significantly increased in Group B (0.46 vs. 0.62) ($p < 0.01$) (Table 5).

### DISCUSSION

In our 6-month prospective examination, the patients in Group A were treated with short-acting β2 agonists as needed and the regular use of low doses of ICS, while the patients in Group B were treated according to GINA with only β2 agonists as needed. As the groups were similar in many parameters at the beginning of the examination, i.e., the only difference being the methods of treatment, the differences that occurred during the examination could be explained by the effects of different therapies.

**TABLE 3**
**PD20, Initially and at the End of the Study**

| | Group A | | Group B | |
|---|---|---|---|---|
| | **Initially** | **End of Study** | **Initially** | **End of Study** |
| $PD_{20}$ μmol | 0.98 ± 0.86 | 2.07 ± 1.36* | 1.04 ± 0.49 | 0.62 ± 0.31** |

*$p$ < 0.001, **$p$ < 0.05.

$PD_{20}$ = provocative dose of histamine causing a 20% fall in FEV1.

**TABLE 4**
**$PD_{20}$, Initially and at the End of the Study when Compared to the Atopic Status**

| | Initially | End of Study |
|---|---|---|
| Group A | | |
| Atopic | 0.92 ± 1.16 | 2.01 ± 1.42* |
| Nonatopic | 1.14 ± 1.16 | 2.21 ± 1.24** |
| Group B | | |
| Atopic | 1.04 ± 0.43 | 0.64 ± 0.30* |
| Nonatopic | 1.03 ± 0.87 | 0.52 ± 0.40 |

*$p$ < 0.001, **$p$ < 0.05.

**TABLE 5**
**The Change in Daily Asthma Symptom Scores and the Use of Rescue Medication[a]**

| | Group A | | Group B | |
|---|---|---|---|---|
| | **Initially** | **End of Study** | **Initially** | **End of Study** |
| Daily asthma symptom score[b] | 0.63 ± 0.14 | 0.30 ± 0.40* | 0.46 ± 0.19 | 0.62 ± 0.13** |
| Day use β2 agonists, puff/week | 1.17 ± 0.24 | 0.51 ± 0.18* | 0.86 ± 0.38 | 1.15 ± 0.35* |
| Night use β2 agonists, puff/month | 2.51 ± 0.76 | 0.58 ± 0.69* | 2.12 ± 0.55 | 2.31 ± 0.57 |

*$p$ < 0.001, **$p$ < 0.01, [a]values represent means ± SEM, [b]daily asthma symptom scores shown as the median sum of daytime and nighttime symptom scores.

The analysis of lung function parameters at the end of the study shows that there was only a significant improvement of FEV1 in Group A, while there was a small, but statistically significant, decrease of FEV1, FVC, and PEF in Group B. After having compared the parameters of lung function between Groups A and B at the end of study, we did not find statistically significant differences. Jatakanon et al. also have found a significant improvement of FEV1 after 1-month treatment with budesonide 800 μg[7]. The unchanged values of other lung function parameters can be explained by the initially high values of these parameters. However, the significant decrease of FEV1, FVC, and PEF in Group B after 6 months may be explained by seasonal variations of asthma and the absence of the profilactic effect of ICS. It may also point out that a delay of the use of ICS in these patients during a long period probably leads to the progression of inflammation and, hence, to a small, but significant, decrease of lung function[8,9]. Bibi et al. came to similar results in their study in which 198 children with intermittent asthma were observed during a 5-year period. They found that in the group treated only with bronchodilators as needed, there was a significant decrease of FEV1/FVC in comparison with the group treated with low doses of ICS, which serves as a marker of the remodeling of airways[10]. This finding supports the hypothesis that asthma patients who do not receive preventive anti-inflammatory treatment have an increased risk of airway remodeling[11].

Bronchial inflammation precedes bronchial obstruction and symptoms in asthma, as well[3]. Patients with mild intermittent asthma have normal spirometric parameters and few symptoms as it was proved in our study; all the patients had FEV1 > 80% predicted. Therefore, the measurement of BHR was chosen as an indicator of bronchial liability.

In this study, the measurement of BHR as a hallmark of inflammation in asthma was the primary outcome parameter of the effects of ICS during 6 months in the patients with mild intermittent asthma who were treated at least 1 year with only β2 agonists as needed. In all the patients, a nonspecific bronchoprovocative test was performed with increasing histamine doses and the provocative dose, which leads to 20% decrease of FEV1 ($PD_{20}$), was found. The received results show a significant impact of ICS on BHR. In Group A, there was a significant decrease of BHR and a decrease of diurnal PEF variability, which was in accordance with the results of Sono et. al.[12] They studied the use of low doses of beclomethasone dipropionate (200 μg) in the treatment of newly detected mild intermittent asthma and concluded that after 8 weeks of treatment, there was a significant decrease of BHR[12,13]. Group B, treated with only short-acting β2 agonists, is characterized by an increase of BHR and a small, but significant, increase of PEF variability after 6 months. The statistically significant difference in PEF variability was found when Groups A and B were compared at the end of the study. The received results support the findings that a delayed use of ICS can make inflammation and BHR worse[14,15,16].

The atopic status and BHR are genetically determined, but it is also well known that BHR is present with both atopic and nonatopic asthma. In his study, Sanfort shows that BHR on metacholine is under genetic control of about 66%[17].

The objective of our study was to analyze BHR initially and at the end of the study in relation to the atopic status. The received results show that at the beginning of the study $PD_{20}$, there was no statistically significant difference between atopic and nonatopic patients, which marks the presence of BHR in nonatopic asthma and points to different mechanisms of its occurrence. Our results are in accordance with the results of Mochiyuki et al,. which show that there is no difference in degree of BHR between atopic and nonatopic asthma patients[18]. At the end of the study in the atopic and the nonatopic patients in Group A, there was a significant decrease of BHR, while BHR remained unchanged in the nonatopic patients, but was significantly increased in the atopic ones in Group B[19].

In patients with mild intermittent asthma, the advantages of ICS have to be weighed against the disadvantages. Although a diminishing effect on airway inflammation has been widely demonstrated, even in patients with mild disease, the impact of inhaled steroids on the long-term prognosis is less clear. The local side effect, such as oral candidiasis, and the systemic side effect, such as adrenal suppression, have been reported with higher doses of ICS. In our study, the patients were treated with low doses of ICS, so there were no systemic side effects and only three patients (3.4%) had oral candidiasis.

A control of asthma symptoms is very important in asthma treatment. Although it does not represent the exact index of airway obstruction, the symptoms point to the patient’s perception of disease progression and, thus, the need for medication. As the patients had intermittent asthma, as-needed therapy was not expected to result in changes in baseline airway function or in asthma symptom scores. Our study shows that at the end of examination, there was a small, but statistically significant, decrease of asthma symptom scores in Group A and an increase in Group B. Furthermore, in Group A, there was a decrease in daily and nightly use of short-acting β2 agonists. In Group B, there was an increase of daily use rescue medications. Our results are similar to the results of Lorentzson et al., who found a significant decrease of nightly asthma symptoms and the use of β2 agonists after treatment with 200 μg budesonide daily, while daily symptoms were improved with higher dose to 400-μg treatment[20]. Molen et al. also show that the use of budesonide in low doses is superior in comparison with placebo for the control of asthma symptoms and the reduction of rescue medication[21]. In Hoshino and Nakamura's study, the improvement of the disease symptoms and the decrease of the rescue medication use occurred after 8 weeks of treatment with budesonide[19].

The results of the study are limited by the short period of the examination, a relatively small number of observed patients, as well as by the fact that patients with intermittent asthma rarely accept medication prescribed for regular use. In this study, the measurement of BHR as hallmark of inflammation was the primary outcome parameter of the effects of ICS in the patients with mild intermittent asthma. However, we were not able to evaluate the effect of ICS on inflammation by examining other markers of inflammation, e.g., the level of NO in exhaled air, which also may be a limiting factor of the study.

The presented results demonstrate that low ICS doses in the period of 6 months led to a significant decrease of BHR, which is an indirect measure of inflammation; an early introduction of low ICS doses may be more beneficial than β2 agonists alone in patients with mild intermittent asthma. However, we expect that we will be able to provide better insight into the impact of anti-inflammatory therapy on the long-term prognosis of mild intermittent asthma patients only after a longer observation of the patients who were included in the study.
